# A qualitative study to explore the role of pharmacists in healthy weight management in adults in Pakistan: current scenario and future perspectives

**DOI:** 10.1186/s12913-020-05419-8

**Published:** 2020-06-15

**Authors:** Muhammad Atif, Sanah Hasan, Irem Mushtaq, Sareema Javaid, Noureena Asghar, Shane Scahill

**Affiliations:** 1 Department of Pharmacy, The Islamia University of Bahawalpur, Bahawalpur, Punjab Pakistan; 2 College of Pharmacy, Ajman University, Ajman, United Arab Emirates; 3 Department of Education, The Islamia University of Bahawalpur, Bahawalpur, Punjab Pakistan; 4 School of Pharmacy, University of Auckland, Auckland, New Zealand

**Keywords:** Healthy weight management, Obesity, Pharmacies, Pharmacists, Barriers, Pakistan

## Abstract

**Background:**

Pharmacists possess significant potential for providing health services to the public when it comes to issues of weight management. However, this practice has not been observed in most parts of the world including low- and middle-income countries (LMICs) such as Pakistan. The aim of this study was to explore the potential role of pharmacists in providing healthy weight management (HWM) services to adults in Pakistan, and the barriers associated with the implementation of this type of role.

**Methods:**

This descriptive qualitative study was set in seven hospitals (public and private) and three chain pharmacies in Lahore, Punjab – a province of Pakistan. Data was collected from in-depth individual interviews with pharmacists (*n* = 19) and medical doctors (*n* = 15). Purposive sampling techniques were applied to recruit both types of study participants. Telephone contact was made by the trained data collectors with the pharmacists to set the date and time of the interview after explaining to them the purpose of the study and obtaining their willingness and verbal recorded consent to participate. Registered medical doctors were recruited through snowball sampling techniques. The sample size was determined by applying the point at which thematic saturation occurred. All interviews were audio-recorded and transcribed verbatim. The data were analyzed to draw conclusions using inductive thematic content analysis.

**Results:**

Through inductive qualitative analysis eight themes emerged; potential role for community pharmacists, collaborative approaches, barriers, ideal pharmacist-based weight management program, professional requirements and need for training, potential for implementation, current scenario in pharmacies and level of trust of pharmacists. The first six themes were common to both pharmacists and medical professionals. The unique theme for doctors was the ‘level of trust of pharmacists’, and for the pharmacists was the ‘current scenario in pharmacies’.

**Conclusion:**

The majority of participants in our study had strong convictions that Pakistani pharmacists have the potential for provide effective HWM services to their communities. Of concern, none of the participating pharmacies were offering any sort of weight management program and none of the medical professionals interviewed were aware of HWM programs taking place. Medical doctors were of the opinion that pharmacists alone cannot run these programs. Doctor participants were firm that after being adequately trained, pharmacists should only carry out non-pharmacological interventions. To implement a HWM pharmacy model in Pakistan, it is necessary to overcome barriers outlined in this study.

## Background

The World Health Organization (WHO) defines being overweight and obesity as having abnormal or excessive fat accumulation in the body that presents a risk to health [1]. Obesity is a complex, multi-factorial chronic condition that develops from an interaction between genetic and environmental factors [2]. In terms of Body Mass index (BMI), an adult who has a BMI between 25 and 29.9 kg/m^2^ is defined as overweight, while an adult who has a BMI of 30 kg/m^2^ or higher is considered to be obese [3, 4].

Being overweight and/or obese poses a major global public health challenge that is escalating [3]. According to one estimate, worldwide 1 billion people are overweight and more than 300 million people are obese [5]. Obesity can lead to serious health consequences such as hypertension, diabetes, some cancers, coronary heart disease, gallbladder disease, osteoarthritis, gastro esophageal reflux and sleep apnea [6]. Excess body weight accounts for 44% of the global burden of diabetes, 23% of ischemic heart disease and 7–41% of some cancers [5]. Approximately 300,000 deaths occur per year directly attributable to obesity, making obesity the second most common preventable risk factor for mortality after smoking [7]. In Pakistan, approximately 26% of women and 19% of men are obese according to WHO guidelines [8]. Obesity is more prevalent in urban than rural areas of Pakistan [9].

Obese individuals may also suffer from social stigmatization and discrimination [10]. At the same time, obesity and its related health problems pose huge economic impact on health systems and preventing obesity will lead to cost savings [11]. The medical costs associated with obesity include both direct and indirect costs [12]. A study conducted in the United States found that obesity increases overall healthcare costs by 36% and medication costs by 77% [13]. In the US, being obese and overweight is associated with an increase in the average cost of inpatient and ambulatory patients of $395 per annum and $125 per annum, respectively [13]. Many countries have been struggling to reduce the ever-increasing healthcare expenditure associated with obesity, making the prevention of obesity a target for health policy-makers in many countries [4, 14].

Healthy eating, active living and weight management programs are offered in a number of developed countries [15–17]. The one-to-one NHS (National Health Service) weight management program in the United Kingdom and the Lifestyle Challenge Program in the US are good examples of programs that comprise diet, physical activity and behavioral modification as their prime components [15–18]. These programs are run by physicians specialized in nutrition, behavioral psychologists and pharmacists. The patients’ recruitment, care and associated physical assessment are primarily the responsibility of the physician. The behavioral psychologist focuses on prevention, addresses health disparities, reduces psychological distress and enhances psychological wellbeing. The role of the pharmacist is to screen patients, review their history, carry out routine assessments and provide medicine-related information to patients [16].

The US lifestyle challenge program developed a set of requirements of a pharmacist-based program for weight loss [16]. The program incorporated diet, physical activity and behavioral modification to lose weight, with special importance given to behavioral therapy. Other studies have also used some of these features including; diet plans, physical activities, self-monitoring, physical evaluation (weight and waist measurements), food diaries; providing motivation and encouragement and goal setting for promoting the adherence of obese people to healthy lifestyles [19, 20].

A study in Scotland [21] outlines the skills that pharmacists needed to acquire in order to effectively take part in HWM services including; consultation skills, estimation of body fat, measurement of cholesterol levels and advice on weight loss products. However, critical evaluation of the efficacy and cost effectiveness of such models of service provision is still lacking. Thus, in a multi-disciplinary environment it is the responsibility of all healthcare providers to identify obese people, counsel and educate them about the risks of obesity, and guide them through an appropriate weight management program.

Community pharmacists in Pakistan offer a range of services that may include: counseling, dispensing of prescription and non-prescription items, supply chain management, provision of medicine-related information to patients, participation in health-promotion programs and diagnostic services by using hand-held devices in the home. Multiple non-pharmacological interventions for managing chronic diseases are also provided by community pharmacies. The aforementioned roles of community pharmacists are in line with the Drug Regulatory Authority of Pakistan (DRAP) Act no. XXI of 2012 [22]. Similar to what is offered in developed countries, in emerging nations such as Pakistan effective interventions to tackle the obesity epidemic by involving the integrated efforts of doctors, nurses, dieticians, psychologists and pharmacists is urgently needed.

Several studies from more developed countries [23–27] have examined the potential role of community pharmacists in providing preventative and management services for tackling the obesity epidemic. However, very few studies [21, 28, 29] have determined the barriers that hinder the successful implementation of such services. To achieve functional systems that are effective in providing HWM programs, pharmacists should be well equipped and sufficiently trained to undertake this role [28, 30]. Moreover, the level of success of pharmacists providing obesity management services depends upon the acceptance of both physicians and consumers of these pharmacy services.

Clearly, pharmacists have an important role in delivering HWM programs, but this is not yet happening in Pakistan. Therefore, it is unknown what the barriers to providing such services might be. This needs to be understood before embarking on such activities. In light of this, the aim of this study was to explore the *potential role* of pharmacists in HWM in adults and the barriers to implementation in the context of Pakistan. The focus was on determining the acceptability by doctors and readiness of community pharmacists regarding the implementation of a pharmacist-based weight management service.

## Methods

### Study design

A descriptive qualitative study design was employed as an exploratory strategy [31] in which in-depth, face-to-face individual interviews of approximately 30 min duration were conducted by SJ and NA with community pharmacists and medical doctors. Medical doctors who were general practitioners, endocrinologists, gynecologists, orthopedic surgeons and cardiologists were included in the sampling frame as they encounter obese individuals in their routine clinical practice.

### Study setting

The study was conducted in Lahore, which is the second largest city in Pakistan with a population of approximately 9 million people. There are 16 public sector and 42 private sector hospitals in Lahore. Both independent and chain pharmacies provide medicines and other medical products to meet the needs of the public. Most of the private community pharmacies in Lahore are largely “drug stores” where availability of a community pharmacist within the premise is limited. However, many well-established chain pharmacies employ registered pharmacists to provide value-added clinical services.

### Sampling technique and data collection

Data were collected between 31st January and 31st March 2016. A purposive sampling technique was applied to recruit the study participants. The final sample size was determined by the likely point at which thematic saturation was achieved during the data analysis phase and the accepted size of qualitative studies in the literature [32, 33]. To recruit community pharmacists, the landline number of various chain pharmacies, selected at random, were obtained from the telephone directory. Contact via phone was then made with the pharmacists to set the date and time of the interview after explaining the purpose of the study and confirming their willingness and oral informed consent to participate. Medical doctors registered with the Pakistan Medical & Dental Council (PMDC) were recruited through convenience sampling and snowball techniques [34]. The Doctors were selected from sub-specialities that are impacted on by obesity including: general practice, orthopaedics, endocrinology, cardiology, gynaecology and nutritional doctor.

Before starting the interviews, rapport was established with the consented participants and they were interviewed at a convenient place that was comfortable for them, as mutually agreed by both interviewer and interviewee. Their verbal consent to participate was also captured in the audio-recording.

All interviews were conducted in Urdu, the national language of Pakistan by SJ and NA. The interview guide was developed through identifying gaps in the literature and was designed to answer the overarching research questions. Before conducting interviews with the recruited study participants, the draft interview guide was pilot tested to ensure uniformity and face validity with a medical doctor and a pharmacist to ensure that the questions were understood as posed. Their data was not included in the study. This process took approximately 35 min per interview. The interview guide was modified accordingly, resulting in a comprehensive and unambiguous set of semi-structured questions with sound face validity [35]. The interview guide is located in Supplementary File 1.

All interviews were audio recorded and transcribed verbatim in Urdu, and field notes were taken during the interviews after obtaining permission from the participants. Written transcripts were compared with the recorded interviews to ensure accuracy. Thereafter the transcripts were translated into English for thematic analysis. Study participants were able to listen to their own recorded interview or read the written transcript if they wished [36, 37]. As part of this member checking process, no participants took up this opportunity nor provided feedback on any of the transcripts.

### Data analysis

A manual coding process was used (as opposed to software enabled analysis) with data being analyzed through general inductive thematic techniques [38] as follows. First transcripts and field notes were read a number of times by SJ and NA to allow familiarization with the content of the data. Each transcribed interview was independently coded and analyzed by SJ and NA [39]. The manual data coding process then began. This involved going through the transcripts and highlighting with underlying passages of meaningful text and ascribing them to categories. By going through one transcript after another, categories were built-up (inductively) from the data. Thereafter the final thematic content was read several times to identify major themes and link them with more abstract categories. The emergent themes were then argued and defended through peer-debriefing by the research team in formal meetings to reach a final consensus and ensure credibility.

For the purposes of this paper when describing the findings, ‘most’ refers to themes endorsed by at least 75% of the pharmacist or the physician participants; ‘many’ refers to those made by half to under 75%; ‘some’ refers to ideas noted by 25% up to half of a group; and ‘few’ to concepts noted by fewer than one-quarter of a participant group.

### Ensuring rigour

This qualitative study followed the principles of research rigour laid down by Lincoln and Guba (1985). Credibility or confidence in the truth of the findings are their interpretation came about through peer-debriefing during the analysis phase where researchers met to argue and defend the emerging themes. Member checking is another process that is expected to improve the credibility of qualitative studies. This involves transcript checking by participants. Unfortunately, none of the participants took up this opportunity.

## Results

A total of 33 participants were interviewed, which makes for a reasonably sized qualitative study. Thematic saturation was reached after conducting interviews with 18 pharmacists (22 invited, four refused to participate) and 15 doctors (21 invited, six refused to participate). Among the pharmacists, 15 were male and three were female and for the medical doctors, 10 were male and five were female. The average interview duration was 25.5 min (± SD 3.4) for pharmacists and 19.9 min (± SD 2.8) for doctors. Respondent demographics are outlined in Table 1.

**Table 1:** Characteristics of the respondents

Pharmacists			Medical doctors			
Respondent	Gender	Interview duration (minutes^a^)	Respondent	Gender	Specialization	Interview duration (minutes^a^)
**A**	Female	27	**A**	Male	GP	21
**B**	Female	29	**B**	Male	GP	20
**C**	Male	20	**C**	Male	Orthopaedic	24
**D**	Male	29	**D**	Female	Gynaecologist	19
**E**	Male	23	**E**	Male	Cardiologist	22
**F**	Male	25	**F**	Female	Nutritionist	16
**G**	Male	27	**G**	Male	GP	21
**H**	Female	26	**H**	Male	Cardiologist	18
**I**	Male	21	**I**	Male	GP	23
**J**	Male	26	**J**	Male	Endocrinologist	16
**K**	Male	27	**K**	Male	GP	20
**L**	Male	28	**L**	Female	Nutritionist	18
**M**	Male	31	**M**	Female	Endocrinologist	25
**N**	Male	24	**N**	Male	Orthopaedic	17
**O**	Male	26	**O**	Female	Gynaecologist	19
**P**	Male	28				
**Q**	Male	24				
**R**	Male	18				

^a^Rounded, GP =  General practitioner

Analysis of data yielded eight emergent themes which are summarized in Table 2. Six themes were common to both pharmacists and doctors. One discrete theme emerged from the pharmacists’ interviews (theme 1) and doctors’ interviews (theme 8) that weren’t found to be common to both types of professional.

**Table 2:** Summary of themes and category content

Sr. No.	Themes	Categories	
		Pharmacists	Medical doctors
**1**	**Current scenario at pharmacies**	No specific weight management programs were run by pharmacists at their pharmacies.	Not applicable
		Routine measurements such as weight, body mass index, blood glucose level and blood pressure are the services currently delivered by most pharmacies outside of formal programs.	
		Pharmacies are visited by the community for the sake of checking their weight.	
		Programs delivered by pharmacists are appreciated and utilized by high income classes than low income.	
**2**	**Potential role of community pharmacists**	Pharmacists could have a major role in counseling and educating the community.	Pharmacists could not only measure baseline parameters of obesity, but may also advise on lifestyle modification.
		Pharmacists have the potential to be an integral part of weight management programs.	
		Providing weight management services sits under the legal scope of pharmacists’ practice.	Legal scope of pharmacy practice is not known by Dr’s.
**3**	**Collaborative approaches**	Pharmacists alone cannot run these weight management programs.	Pharmacists alone cannot run these programs.
		Collaboration with other healthcare providers, pharmaceutical companies and owners of pharmacies is important.	Collaboration with other healthcare providers is absolutely necessary.
		Government support and collaboration is essential because it is really difficult to run these programs without the support of government.	
**4**	**Barriers**	Low wages, lack of a robust community pharmacy sector and deficiencies in pharmacy education are the major barriers.	Lack of awareness of the professional roles of pharmacists and insufficient facilities at pharmacies are the main obstacles to implementation as seen by Dr’s.
		Owners of pharmacies are not interested in these types of programs because owners focus only on business.	
			Current pharmacies are identified as medicine selling points rather than clinical service providers.
		The government and other bodies that govern pharmacy do not support nor collaborate with pharmacists.	
		The community does not have a clear understanding of the professional roles that pharmacists could undertake.	
**5**	**Ideal pharmacist-based weight management program**	Separate space in a pharmacy is required to implement this program.	Only non-pharmacological approaches should be included in programs.
		Lifestyle modifications should be the core component.	Follow up visits should be planned.
		Robust systems for record keeping and follow-up should be included in the program.	Outcomes should be assessed through measurement of baseline parameters of obesity.
		Assessment of baseline parameters is also essential.	
			Counseling should be tailored for every visit with non-responders.
**6**	**Professional requirements and need for training**	Pharmacists can become professionally competent if they are properly trained and have adequate knowledge to deliver weight management services.	Pharmacists need specialized training about weight management programs to become professionally sound.
		Separate space in pharmacies is required for the implementation of these services.	Basic knowledge about obesity, co morbidities and linking to counseling points should be included in the training sessions.
		Pharmacists are willing to take part in sponsored training sessions in order to update their clinical knowledge in this area.	
**7**	**Potential for implementation**	If governing bodies support pharmacists and collaborate with them to improve the system and processes, then it will be possible to implement these programs.	By creating awareness about the positive image of pharmacists, these programs can be implemented.
		Awareness about the professional roles a pharmacist can play needs to be increased amongst the wider community.	These programs can be implemented if the current system is improved.
			Current burdens on doctors can be shared through implementation of these services.
		More practical training sessions should be included in the undergraduate pharmacy curriculum.	
**8**	**Level of trust on pharmacists**	Not applicable	Pharmacists are capable to be a part of the delivery of weight management programs.
			They can only deliver non-pharmacological components related to these programs.

Note: There were no differences within each professional group and so we have presented pharmacist versus medical doctor views as two large groups of participants

### Theme 1: current scenario at pharmacies

Pharmacies weren’t offering formal HWM programs and this is a new concept to introduce within the pharmacy sector in Pakistan.

*“No, I do not think so that it exists anywhere in Pakistan.”* (Pharmacist Q)

*“Pharmacist-based weight management programs are a new concept in Pakistan”* (Pharmacist J)

Despite the importance of reducing chronic disease, demand for weight management services by the community is generally low according to the pharmacist respondents. Some participants stated that the demand depends upon the geographic area of the pharmacy. People from elite class areas and those who are educated usually demand these services. Moreover, foreigners and those people who have visited abroad also demand these types of services:

*“People who come from abroad demand these sorts of services from pharmacy, but such people are few in number. It also depends upon the area in which your pharmacy is situated, for instance, if you are working in DHA (high income population) then, of course, demand is more than if pharmacy is situated in some area like Shad Bagh (low-middle income population)”* (Pharmacist D)

### Theme 2: potential role of community pharmacists

The majority of pharmacists had similar views about the potential for a positive role in HWM at community pharmacies. They considered that although there was no community pharmacy set up to deliver HWM programs in Pakistan, if initiative was taken to implement these services then pharmacists could be trained to play a key role in counseling and support, and increase awareness about the importance of weight management and these types of services. Counseling about lifestyle modification was regarded as a pivotal service a community pharmacist could provide:

*“Community pharmacists are not currently adequately trained about HWM program but if the pharmacists are properly educated, then they can play a very positive role in this aspect.”* (Pharmacist F)

The majority of pharmacists considered themselves to be capable of providing HWM services; however, one participant had doubts and stated that pharmacy students are not sufficiently trained at the undergraduate level and therefore lack the expertise required to deliver these services:

*“That’s something we need to look at. Normally students are not given awareness, not told about it so when a pharmacist joins* [a pharmacy or shop]*, he is not that expert.”* (Pharmacist M)

Most pharmacists interviewed also thought that providing weight management services fit within their legal scope of practice: *“Of course, this is in the legal scope of the pharmacist. And this is in the laws that pharmacists can provide these services.”* (Pharmacist I).

Medical doctors considered measurement of baseline parameters for obesity, lifestyle modification, counseling and creating awareness among community-dwelling customers as possible roles of a community pharmacist:

*“Pharmacists can assess basic parameters of obesity. They can counsel the patient and make the patient ready to think over all these things -- i.e., follow up, nutritional changes and behavioral changes.”* (Medical doctor A - GP)

One respondent considered the pharmacist as a moderator between patients and medical doctors (*“We need a moderator between doctor and patient.”)* (Medical doctor B - GP).

### Theme 3: collaborative approaches

Collegial approaches and teamwork were recognized as prerequisites for implementing pharmacist-led weight management services by the majority of pharmacist respondents. Most pharmacists agreed that everyone on a healthcare team should work together, putting aside their sense of self-importance and professional boundaries:

*“I think we should collaborate with other healthcare professionals. Every person who is involved in patient care should work together to bring weight management services in community pharmacies. We should not indulge in things like, ‘if I am a doctor then I will not work with the pharmacist or nurse’. Although these things are there, as healthcare professionals our ultimate purpose is patient care and safety, so we have to work together for that.”* (Pharmacist C)

The majority of pharmacists also considered government collaboration a necessity for implementation of HWM services: (*“Obviously nothing can happen without government’s collaboration.”)* (Pharmacist P).

Most medical doctors also agreed that teamwork among all healthcare professionals is needed for the success of such a service. They thought it would be difficult for pharmacists to implement the services alone without the support of doctors (*“It is a task of well-defined team. It cannot be possible without collaboration. In my opinion he should play his role in collaboration.”)* (Medical doctor K - GP).

However, two doctors thought that pharmacists could not conduct these roles even in collaboration with other health professionals and pharmacists are not the right professionals to deliver HWM services (*“In my opinion, mainly the dietician and the physiotherapist should be the stakeholders.”)* (Medical doctor I - GP).

### Theme 4: barriers to service provision

The majority of pharmacists interviewed stated that improper community pharmacy set up, lack of a purpose-build space and poor collaboration from governing bodies such as Drug Regulatory Authority of Pakistan, provincial and district drug control units etc. are the major barriers to implementing weight management services in community pharmacies. The concept of the community pharmacy is not well established in Pakistan, and often is seen as just a place to sell medicines (*“We cannot say that the pharmacies in our country are community pharmacies; they all are just medicines’ sales points and nothing else.”)* (Pharmacist C).

Many of the pharmacists also explained that their current wages are too low to encourage them to provide extra services such as weight management. One respondent revealed that some pharmacists had actually quit their profession for this reason (*“You know some people drop their field just because of low wages so our authorities need to think about it.”)* (Pharmacist O) Some revealed that lack of support for pharmacists from governing bodies or their pharmacy professional bodies and associations as another major barrier ( “*… there are pharmacists who want to work* [to provide additional services] *but authorities do not allow them.”)* (Pharmacist R).

Another barrier noted by the majority of pharmacists is the lack of respect for the pharmacy profession held by the general public who saw pharmacists as sales oriented (*“Basically, everyone is thinking of business. You can see that the owner standing here only wants an increase in sales because this is the ultimate goal. They do not want anything else, they need sales.”)* (Pharmacist K).

Most pharmacists pointed out there is a gap between what pharmacists learn in their formal training and their actual practice, which negatively impacts pharmacists’ capabilities to implement clinically-oriented services:

*“Well they are capable but need grooming because there is a lot of difference in our education system and a real professional life. Whatever we have been taught in our education system is not the case according to the professional world.”* (Pharmacist C)

In contrast, most of the medical doctors explained that the general public did not understand what pharmacists are licensed to do and so those with low levels of education or health literacy were unlikely to accept such new pharmacist-run services as a healthy weight control programs:

*“Our literate class will accept these services because it is all about awareness. But people from poor socioeconomic background and illiterate people will hardly accept these kinds of things.”* (Medical doctor B - GP)

Most of the responding medical doctors also cited the lack of adequate facilities, absent pharmacists during opening hours and the business-orientation of the community pharmacy practice as barriers to implementing weight management services (*“There are insufficient facilities at our current pharmacies. In simple words, I would not be wrong if I said that there is nothing at our current pharmacies except medicines.”)* (Medical doctor G - GP).

### Theme 5: ideal pharmacist-based weight management program

Most pharmacists agreed that the crucial components of an effective weight management program include: lifestyle modification counseling, proper equipment to measure obesity-related parameters, separate space where the services would be conducted in private and encouraging participants to attend timely follow-up visits. In addition, a few respondents emphasized the importance of proper record-keeping to successfully run the program:

*“I think there should be proper lifestyle modification counseling and there must be a separate area within the pharmacy for this. It should also include proper equipment for weight measurement, cholesterol measurement and blood pressure measurement; and it should also include set targets for outcomes so that the progress of the program can be assessed.”* (Pharmacist A)

Medical doctors also identified non-pharmacological components like behavioral and lifestyle modification counseling, flexible follow up visits, assessment of outcomes through measurement of baseline parameters for obesity and goal setting as key elements in an ideal pharmacist-led weight management program (*“Follow up should be there but number of visits should not be high; I think this is hectic for the patient. Six months follow-up is enough for a patient.”)* (Medical doctor H - Cardiologist).

### Theme 6: professional requirements and need for training

Well-trained staff with a sound knowledge of, and training in the subject, a designated space within the pharmacy and government support and collaboration were the professional requirements identified by most respondents:

*“Professional needs include that each and every person working in this pharmacy should be trained and be sincere in their work. They all need grooming. The pharmacist should be trained enough. In addition to* [knowing] *the names of medicines, a pharmacist should be capable of guiding* [people] *about other perspectives, for example, lifestyle modifications. There should be some kind of software carrying the detailed outline of the project. There should be a separate corner in the pharmacy which can be used as an area where such services can be given.”* (Pharmacist C)

Most pharmacists also wanted the training to be compulsory for better outcomes but felt they should also be funded because of their low wages ( “*… you know, we do not have that much earning. Such workshops/trainings always have very high registration fees so it is not easy to pay.”)* (Pharmacist L).

Most medical doctors were also of the view that specialized training pertaining to HWM is of pivotal importance for pharmacists who should be trained to counsel people about diseases related to obesity, ways of classifying levels of obesity and how to measure the parameters linked to obesity. Some doctors thought it was necessary to include knowledge of complementary and alternative medicines in the training as well:

*"First of all they should know the classification of obesity. Then, different measurements of weight, abdominal circumference and body mass index should also be included in the training sessions. A pharmacist should know the basic knowledge of obesity and disease related to obesity e.g., hypertension, diabetes etc. They should also know at what step they need to involve a physician.*

*"They should know the basics of complementary alternative medicines as well, but should deliver only in specific circumstances."* (Medical doctor N – Orthopaedic)

### Theme 7: potential for implementation

According to the pharmacists, HWM programs can be implemented in the future after incorporating changes to the system. Most pharmacist participants identified the governing bodies as an important backbone for implementing such programs. They viewed government policy-makers as having the power to pursue and support this new concept and to change the negative perception that community members have towards pharmacists (*“Perception about the pharmacists is still old-fashioned among the community, so it is the duty of the government to promote activities including pharmacists so that the interaction and trust between the people and pharmacists can be enhanced”.)* (Pharmacist A).

Some pharmacists stated that heightened awareness about preventive health programs such as HWM can be created through campaigns mounted by pharmaceutical companies and by educating the community through seminars (*“I think if we give awareness to people then they will welcome us. If we start from campaigns, then it can be implemented”.)* (Pharmacist H).

They also held the view that the undergraduate pharmacy curriculum should be thoroughly revised to meet the new challenge of preventing non-communicable diseases through controlling obesity. Some participants suggested creating a model pharmacy in all academic institutions so that students can be trained to engage with such programs (*“I think there should be a model pharmacy in every (academic) institution so that students can practice there and do not feel much gap when they enter in their professional careers.”)* (Pharmacist J).

The medical doctors agreed that it would be possible for pharmacists to gain the trust of the community by creating awareness among people about the positive role beyond just filling prescriptions or selling medicines that they can potentially fulfill ( “*… .these can be applied in Pakistan only with certain special conditions, for example, if the people are motivated, counseled or given awareness related to it.”)* (Medical doctor A - GP).

Some medical doctors also stated that if HWM-related services were to be provided by pharmacists, then physician burden would be greatly reduced, freeing them up to provide more directly needed medical services: (*“If pharmacists will be performing such roles, then the burden on doctors will surely be decreased so it will be helpful to the community.”)* (Medical doctor G - GP).

### Theme 8: level of trust for pharmacists

Most medical doctors said they trusted pharmacists to deliver HWM programs and considered the pharmacist to be a competent and qualified healthcare professional. They suggested that pharmacists could deliver non-pharmacological interventions to patients on their own but should consult doctors for pharmacological interventions. However, some medical doctors considered that a trained and qualified pharmacist could deliver all possible interventions (*“They are competent enough. If they have weight management services in their pharmacy, then, they can perform these roles very well.”)* (Medical doctor D - Gynaecologist).

In contrast, two medical doctors thought that pharmacists have no role to play in helping people manage their weight *(“Disease related obesity cases should be referred to physicians.”* (Medical doctor M - Endocrinologist).

This range in responses may be related to the fact that most medical doctors were not aware of the legal scope of pharmacy practice, although two physicians incorrectly stated that providing HWM services is not covered under the legal scope of pharmacists.

## Discussion

This study set out to explore the perceptions of the role of pharmacists in weight management in the context of Pakistan as a low and middle-income country. It aimed to understand the extent to which services were being provided but more importantly the barriers to doing so. The findings indicate that none of the responding pharmacists working in community pharmacies of Lahore, Pakistan, offered HWM programs although the potential to do so was there. Most of the pharmacists and medical doctors participating in this study were in favor of this value-added clinical role for pharmacists but the doctors felt pharmacists couldn’t deliver the service alone. This finding is consistent with several international studies. One recent study conducted in Scotland reported community pharmacists successfully provided weight-loss medications and advice about their use, information related to physical activity and diet, calculated BMI and offered numerous other services related to HWM [21]. Likewise, an earlier study from the United Kingdom (UK) demonstrated a HWM program working well in community pharmacy and that the program increased the opportunity to identity other pharmaceutical care needs of patients and help establish the role of pharmacists in the management of obesity [40]. It is hoped that this will also occur in Pakistan. Along the same lines as the UK study, an Australian study also reported that pharmacists possess a unique role in providing these services to the public [41].

Our findings clearly demonstrate that although Pakistani pharmacists strongly believe they could play a vital role in delivering HWM programs and thereby help improve population health outcomes, this can only be accomplished if a collegial relationship exists amongst healthcare professionals. This finding resonates with the outcome of another HWM study [42] that recommended building a program based on a holistic approach, incorporating various healthcare professionals including doctors, dieticians, physiologists, psychologists and pharmacists, in order to improve healthcare outcomes for obese patients. A practical example of this holistic approach is the lifestyle challenge program in the U.S. in which a multidisciplinary team, co-directed by a pharmacist and a doctor, effectively improved the quality of life of obese people with significant reduction in their weight [16, 25].

Interestingly, two medical doctors in the present study stated that pharmacists should not play a role in HWM services, even with the collaboration of other healthcare professionals. This was in line with the findings from another Pakistani study which reported that although doctors considered pharmacists to be “drug experts”, their expectation of pharmacists as providers of quality clinically-focused pharmacy services is low [43]. The wider pharmacy HWM literature does not report the under confidence of medical doctors as a barrier to implementation [40, 44–46] and so perhaps this patch protection of multidisciplinary roles is specific to Pakistan and may be part of the culture of the health care system.

Most of our non-pharmacy study participants were not familiar with the legal scope of pharmacist practice and so did not know whether pharmacists were allowed to provide HWM services. This runs counter to the position of the American Society of Health-System Pharmacists [39] and the UK National Health Service (NHS) [19, 23] which encourage pharmacists to educate obese individuals about weight loss and reduce the impact of non-communicable diseases through offering smoking cessation and obesity management programs.

The pharmacists and doctors in our study identified a number of barriers preventing pharmacists from offering HWM programs for community-dwelling residents. Some of these alignment with the international literature and include: the lack of proper facilities and training, low wages of community pharmacists, the business-oriented attitude of the pharmacy owners, the absence of support from the government, the lack of awareness among the public about the role of pharmacists and the lower status of pharmacists in relation to physicians [40, 44, 46]. It is interesting that many of the barriers identified in our study in Pakistan (an LMIC) are also manifest in studies on community pharmacies in the UK, Australia and New Zealand. These studies reported that deficiencies in the infrastructure of pharmacies, the limited number and overburdened nature of community pharmacists, and financial limitations as key barriers to community pharmacists successfully providing HWM services [20, 21, 29, 47]. Likewise, a study conducted in Texas identified the lack of reimbursement to pharmacists as another major obstacle [23, 28]. Our study also highlighted the fact that HWM programs would be more successful if government and health policy-makers collaborate with pharmacists. An Australian study emphasized that the expertise of pharmacists and their accessibility to the general public who may require weight management was mostly overlooked by health policy-makers [25]. It is recommended that health policy-makers in Pakistan should recognize the barriers identified in this study that are currently faced by pharmacists and help to shape the model pertaining to HWM.

A major barrier identified in our study was the lack of understanding of the pharmacist role by the public and this is an issue in high income countries as well [40, 44, 46]. The public do not expect or look for HWM programs offered in community pharmacies in Pakistan. Peletidi & Kayyali (2019) highlight that training, talking to patients (the approach taken) and too little advertising has resulted in poor sustainability of the HWM program in community pharmacy in England [46]. Our study parallels the results from a Scottish interview study in which most participating pharmacists indicated that the general public was unaware that community pharmacists could provide HWM services for weight loss [21]. Similar findings in the UK and Australia revealed that pharmacists are not a preferred source of information for weight loss [40, 47]. However, it was reported in another study that once the general public received such advice from pharmacists, even if they had not initiated a request for it, the general public agreed that it was beneficial [47]. This appears to be a global issue not only in developed nations but in those low and middle-income countries such as Pakistan where both the doctor and pharmacist workforces are stretched beyond the imaginable.

Another barrier is that Pakistani pharmacists are not currently educated and trained to offer HWM services and again this appears to be a similar issue in high income countries [44, 46]. Although pharmacists are considered one of the most trustworthy and accessible health care professionals in developed nations [44] and are well situated toward community service provision competence remains an issue in Pakistan [48, 49] but also in Australia [20, 25]. Due to the absence of formal training, the pharmacists in our study were not confident in providing HWM services, a finding that is echoed in interviews with Texas-based community pharmacists [50]. Other studies have also noted that although pharmacists were keen to provide HWM services [25]. They also believed that these services could greatly benefit the community, even though they were not routinely providing these services [50]. This was mostly due to the lack of confidence in their ability to provide such a program [50]. The up-skilling of pharmacists would not only result in better outcomes, but should also increase their confidence and comfort in the counseling of obese patients [28, 50]. These studies point to the need for expanding the roles and skills of pharmacists, either by incorporating HWM information in pharmacy curricula or by providing targeted short courses to train practicing pharmacists.

The pharmacists in this study suggested that the ideal pharmacy-led program should include suitable lifestyle modification counseling, proper equipment to measure obesity-related parameters, separate space for these services, timely follow-up visits by participants and proper record-keeping to successfully run these programs. Similarly, the physician participants recommended incorporating behavior modification counseling, goal setting, flexible follow up visits and assessment of outcomes through measurement of baseline parameters for obesity.

### Implications for policy-makers, regulators and practitioners

This is a relatively large qualitative study and as far as the authors are aware, the first of its kind to explore extended clinical service provision in community pharmacy for healthy weight management in Pakistan. This study investigates the views of both Pakistani pharmacists and doctors. The Pakistani community pharmacy sector is aiming to gain traction in the provision of clinical services and this study adds weight through gaining the views of pharmacists and doctors in the country. Further, it highlights the barriers that must be addressed and the support and collaboration required by government, policy-makers and regulators to strengthen the Pakistani health system and help enact these services. This must include better training and relief from busy dispensing schedules to ensure pharmacists have the time to properly implement these services. In high income countries national policy dictates the remuneration packages that help to facilitate service provision. This model may also benefit the implementation and sustainability of HWM in Pakistani community pharmacy. The government also may have a remit (along with professional pharmacy bodies) to increase awareness with the public of the importance of community pharmacy assisting with these types of public health measures.

This study suggests implications for pharmacists involved with implementing clinical services in community pharmacy in Pakistan. For pharmacists there is a need to undertake more training, overcome their lack of confidence which is also reported in high income countries. Training will help the approach that pharmacists take and likely increase public awareness [46, 48]. A national campaign to make medical doctors more aware of the role of pharmacists in public health initiatives would be expected to ease the barriers to implementing services in pharmacy and may also help greatly with public assurance that pharmacists are in a good position to do this.

### Limitations

In terms of limitations, this is a qualitative piece of work and participants in our study were from a single province in Pakistan. The qualitative approach was chosen to gain a deep and rich understanding of the situation prior to moving to a more generalizable and large representative quantitative study [51]. As such participants are sample purposively [52] and their views may differ from other reasons and so the results should not be generalized more widely within Pakistan nor to other like-health systems or countries. In other regions the perceptions of the public may be different, the service provision in community pharmacy may vary and the views of medical doctors may not be the same – they may be completely unsupportive or overly positive towards implementation of HWM programs in pharmacy. Having said this the authors do not expect a huge difference across the nation and this exploratory qualitative study provides some indication of what is happening in one province of Pakistan. There is a requirement for large and prudent quantitative studies to confirm the national-level situation.

### Implications for future research

Research needs to be conducted more widely and if the findings are similar, then a national policy could be developed based on research evidence to address this issue head-on. In addition, because a uniform health policy is implemented in Pakistan and medical graduates (pharmacists and medical doctors) from all provinces are working throughout the country, it is expected that the practices in other provinces could be similar to those reported in our study. This will remain unknown until a national level survey is conducted. The perceptions of patients, their families and also policy-makers were not taken into account in our study and this is an area of future research that needs to be undertaken. This could begin with focused qualitative interview based work and then expand into a large sample national consumer panel-based survey in Pakistan.

## Conclusions

The majority of participants in our study strongly believed that pharmacists have the potential to provide HWM services to Pakistani communities provided they are adequately trained to do so. None of the participating pharmacies were offering any sort of weight management program and none of the medical doctors interviewed were aware of such programs taking place. Doctors were generally supportive but suggested that pharmacists alone cannot run the program and that they should only carry out non-pharmacological interventions relating to weight management. There are barriers to the implementation of HWM which are not dis-similar in parts to those outlined in the literature from high income countries. In Pakistan, all stakeholders including the general public, other healthcare providers, regulatory bodies, policy-makers and pharmacists themselves need to be involved in shaping the role of the community pharmacist to deliver healthy weight management programs.

## Supplementary information

**Additional file 1.** Interview protocol

## Data Availability

The raw data on which conclusions of this manuscript rely is available upon request. Please contact Muhammad Atif at pharmacist_atif@yahoo.com.

## Abbreviations

BMI: Body mass index
HWM: Health weight management
NHS: National Health Service
PMDC: Pakistan Medical & Dental Council
UK: United Kingdom
US: United States
WHO: World Health Organization
