# Supplementary material for: A qualitative study to explore the role of pharmacists in healthy weight management in adults in Pakistan: current scenario and future perspectives
Source: BMC Health Serv Res. 2020 Jun 15;20:541. doi: 10.1186/s12913-020-05419-8 (PMC7296957; doi:10.1186/s12913-020-05419-8)
Supplement: Supplementary file 1 — Additional file 1. Interview protocol [file 12913_2020_5419_MOESM1_ESM.docx]

**INTERVIEW PROTOCOL**

| **Discussion Topics** | **Examples of Specific probes** |
| --- | --- |
| **Medical Doctors** | |
| **1. What kind of roles a community pharmacist can play in weight management?** | - Taking part in educational/awareness programs for the community - Selling of weight loss products - Collaboration with other healthcare professionals - Counseling of the patients |
| **2. Do you think it would be legal to confer such responsibilities to pharmacist? If yes then why? If no, why?** | - Do you think it is according to the legal scope of pharmacy practice in Pakistan? |
| **3a. Are you familiar with any pharmacist based weight management program being offered in any pharmacy in Pakistan (We can show you models of different weight management programs running in other countries so you can have an idea)? If yes then please tell us about the details** | - Place where it is delivered - Perceive about the effectiveness of that program - Benefits it hold for the pharmacy - Advantages and disadvantages it holds for the consumers |
| **3b. If no, then what could be the possible reasons that our pharmacies are not having this program?** | - Current pharmacies are not suitable for this program - Pharmacists are not competent enough - Negative feedback from the community - These services are not including in the current goals of our pharmacies - Changes required in our current pharmacies to initiate this program |
| **4. How would you describe an ideal weight management program to be offered in a community pharmacy setting?** | - Key features of the program - Nutritional and lifestyle modification counseling - Assessment of weight, waist circumference, BMI, blood pressure, blood glucose level etc. - Individualized goal settings - What outcomes to assess - Multidisciplinary approach - Stakeholders of the program - Participation of all stakeholders |
| **5. Do you think pharmacists need weight management training courses specially designed according to their needs?** | - Areas of professional skills included in the training session - Length of the program - Format of training courses - Training programs should be compulsory or optional |
| **6. If trained and credentialed, how confident are you that a pharmacist can deliver such a weight management service with those components you have described?** | - Components a trained pharmacist can deliver - Components a trained pharmacist cannot deliver |
| **7. Would you like to add anything else?** | **---** |
| **Pharmacists** | |
| **1. In your opinion what kind of roles a community pharmacist can play in weight management?** | - Taking part in educational/awareness programs for the community - Selling of weight loss products - Should work according to the demands of the consumers - Collaboration with other healthcare professionals - Counseling of the patients |
| **2. Are you familiar with any pharmacist based weight management program being offered in Pakistan (We can show you models of different weight management programs running in other countries so you can have an idea)? If yes then please tell us about the details.** | - Place where it is delivered - Perceive about the effectiveness of that program - Benefits it hold for the pharmacy - Advantages and disadvantages it holds for the consumers |
| **3a. Does your pharmacy offer any type of weight management program? If yes, please elaborate about the key structure of the program** | - Reasons for choosing the program - Consumers’ demands - Any effects on pharmacy e.g. increase in sales - Place in pharmacy to deliver this program - Any special room being allocated - Any specific equipment being used - How did you make yourself competent for delivering this program? - Special training - Part of undergraduate or graduate studies - Methods adopt to upgrade professional skills for delivering this program - Experience gained with the passage of time - Government’s help to set up this program in pharmacy? - How confident do you feel in delivering this program? - Command in delivering the program - Assessment of outcomes of the program - Monitor weight loss - Increase in number of consumers - Consumers’ satisfaction level - Increase in weight loss products demand - Increase in participants’ adherence - Do you think that this program requires any improvement? If yes then what are your recommendations and how will it be beneficial? - What makes your pharmacy suitable for this program? |
| **3b. If no, then what are the reasons?** | - Pharmacy not suitable for the program - Do you think you are not competent enough? If you think that you are competent then what are the other reasons for not having such services in your pharmacy? - Lack of time - Remuneration issues - Negative feedback from community - This is not the goal of your pharmacy - Changes required in the pharmacy to initiate this program |
| **4. In your opinion what professional needs and other requirements are necessary to start a weight management program in a community pharmacy?** | - Well trained staff - Detailed description of programs available - Government collaboration - Infrastructure of the pharmacy |
| **5. How would you describe an ideal weight management program to be offered in a community pharmacy setting?** | - Key features of the program - Nutritional and lifestyle modification counseling - Assessment of weight, waist circumference, BMI, blood pressure, blood glucose level etc. - Individualized goal settings - What outcomes to assess - Multidisciplinary approach - Stakeholders of the program - Participation of all stakeholders |
| **6. Do you think it would be legal to confer such responsibilities to pharmacists? If yes then why? And if no, why?** | - Do you think it is according to the legal scope of pharmacy practice in Pakistan? |
| **7. Are you willing to take part in the weight management training course specially designed according to pharmacists’ needs?** | --- |
